# The *MED13L* Haploinsufficiency Syndrome Associated with De Novo Nonsense Variant (P.GLN1981*)

**DOI:** 10.34763/jmotherandchild.20202403.2021.d-20-00003

**Published:** 2021-04-30

**Authors:** Mateusz Dawidziuk, Anna Kutkowska-Kaźmierczak, Paweł Gawliński, Wojciech Wiszniewski, Monika Gos, Piotr Stawiński, Małgorzata Rydzanicz, Joanna Kosińska, Paweł Własienko, Olga Malinowska Kordowska, Magdalena Bartnik-Głaska, Joanna Bernaciak, Krzysztof Szczałuba, Monika Bekiesińska-Figatowska, Rafał Płoski, Jerzy Bal, Sylwia Olimpia Rzońca-Niewczas

**Affiliations:** 1 Department of Medical Genetics, Institute of Mother and Child, 17a Kasprzaka Street, 01-211 Warsaw, Poland; 2 Department of Molecular and Medical Genetics, Oregon Health and Science University, 3181 SW Sam Jackson Park Road L103, Portland, Oregon, USA; 3 Department of Medical Genetics, Medical University of Warsaw, 3c Pawińskiego Street, 02-106 Warsaw, Poland; 4 Department of Diagnostic Imaging, Institute of Mother and Child, 17a Kasprzaka Street, 01-211 Warsaw, Poland

**Keywords:** MED13L, intellectual disability, loss-of-function mutation, haploinsufficiency

## Abstract

The Mediator complex subunit 13-like is a part of the large Mediator complex. Recently, a large number of patients were diagnosed with mutations in this gene, which makes it one of the most frequent causes of syndromic intellectual disability. In this work, we report a patient with a novel *de novo* likely pathogenic variant c.5941C>T, p.(Gln1981*) in the *MED13L* gene with severe intellectual disability and facial dysmorphism. Uncommon findings like lack of speech, strabismus and self-destructive behaviour present in our patient allowed us to further define the phenotypic spectrum of mental retardation and distinctive facial features with or without cardiac defects syndrome.

## Introduction

Mental retardation and distinctive facial features with or without cardiac defects (MRFACD) syndrome is a type of syndromic intellectual disability (ID) caused by heterozygous variants in the *MED13L* gene resulting in a loss-of-function (LoF), or less often in missense mutations affecting the *MED13L* protein.^1^ It appears that the *MED13L* gene mutations are one of the most frequent causes of ID.^2^ Previously, pathogenic variants of the *MED13L* gene were associated with dextro-looped transposition of the great arteries and ID.^3^ However, additional studies reveal that cardiac disorders are not leading symptoms caused by the *MED13L* gene defects, and both missense and LoF variants can cause MRFACD.^4^ In this study, we present a new case of the MRFACD syndrome in a Polish patient with severe ID, DD and dysmorphic features, which overlap with previous reports, as well as uncommon findings like lack of speech, strabismus and self-destructive behaviour.

## Method

### Informed consent

The study was conducted according to the Declaration of Helsinki Principles. Written informed consent was obtained from the legal representatives of the patient and the study protocol was approved by the Bioethics Committee of the Institute of Mother and Child, Warsaw, Poland (number 30/2012).

### Whole exome sequencing and data analysis

A detailed description of the materials and methods used for this study can be found in the supplementary material of this article.

### Clinical case report

The patient is a 7-year-old boy with severe ID, born to young, non-consanguineous parents after 41 weeks of gestation in an uncomplicated second pregnancy. The first pregnancy had ended by a miscarriage in the 10th week. The patient’s birth weight was 2,300 g (5c<), height 50 cm (25c–50c) and head circumference 32 cm (5c<). His Apgar scores were 7, 9 and 10 points in the 1st, 3rd and 5th min, respectively. He had congenital pneumonia. Brain ultrasound examination performed at the age of 8 months revealed wide lateral ventricles. From the beginning, the child had had axial hypotonia with distal spasticity. He was able to sit at 13 months and walk at about 3 years of age, but his gait was atactic. He presented dysmorphic features such as open mouth with the protruding tongue, wide mouth, high forehead, hypotelorism, biparietal narrowing, sparse hair in the temporal region, discrete telecanthus, bulbous nasal tip, short philtrum, proximal thumbs without ability to catch, first toes curved laterally and an additional nipple on the left side (Fig. S1C–E in Supplementary Material). He had convergent strabismus and astigmatism. At the age of 7, he did not develop speech at all. He had behavioural problems – autoagression (biting hands) and stereotypic movements. Magnetic resonance imaging (MRI) of the brain revealed typical terminal zones of unfinished myelination posteriorly and asymmetric ventricles. The Virchow–Robin spaces were detectable in the same location (Fig. S1B in Supplementary Material). Array CGH (aCGH) taken as primary genetic testing had shown the presence of a 170-kb maternally derived deletion in 22q12.3 (32,323, 842-32,494,686)[hg18]. This deletion encompasses the *LARGE1* gene, in which mutations are responsible for muscular dystrophy-dystroglycanopathy types A and B (OMIM # 613154 and 608840), both inherited in an autosomal recessive manner. The inheritance pattern of the disease and the fact that the mother of the child is healthy indicated that the deletion could be pathogenic only if there was a second mutation in the other allele of the gene.

**Figure S1:**
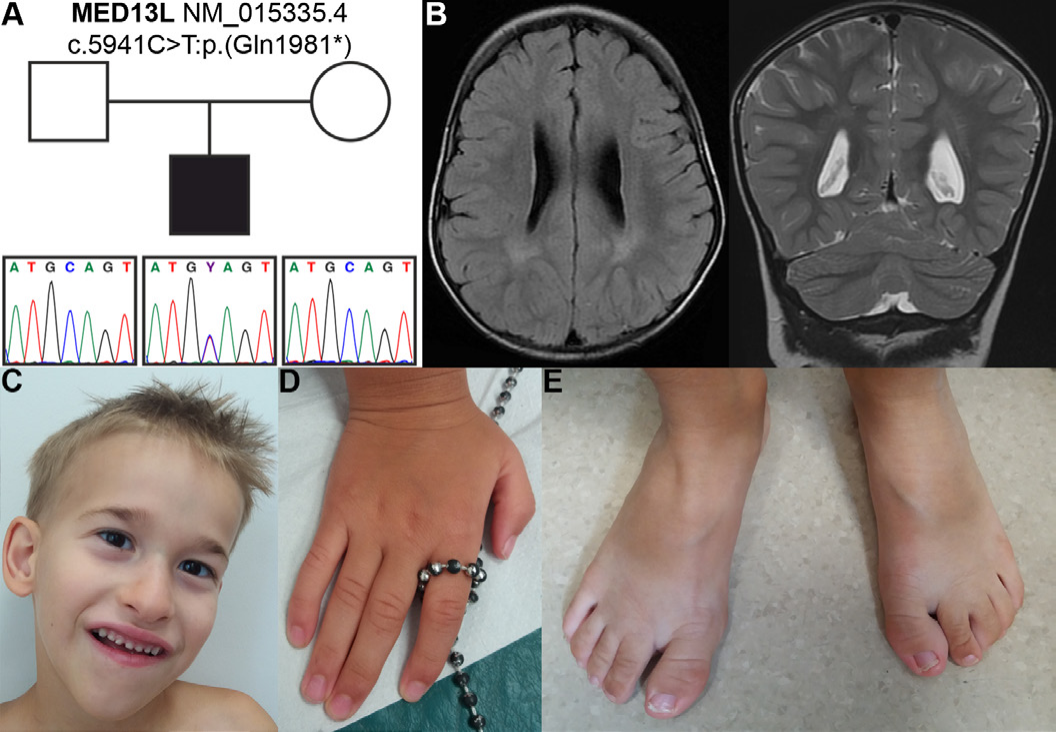
(A) Sanger sequencing confirmed the nonsense variant c.5941C>T in the patient. (B) Axial and coronal brain MRI showing terminal zones of unfinished myelination posteriorly and asymmetric ventricles as well as the Virchow-Robin spaces in the same location. (C) Facial features, (D) hands and (E) feet anomalies in patient at 7 years of age.

## Results

Whole exome sequencing identified a heterozygous single nucleotide substitution c.5941C>T (NM_015335.4) at exon 27 in the *MED13L* gene. This substitution is predicted to cause a premature translational stop codon p.(Gln1981*), affecting the last 229 residues and N-terminal domain of the protein. The variant was not found in the 1000 Genomes, Exome Variant Server and gnomAD databases as well as in our own database. No other phenotypically relevant pathogenic/potentially pathogenic variants were identified in our patient.

*In silico* analysis performed with MutationTaster indicated that the c.5941C>T substitution is a disease-causing variant and is predicted to cause nonsense-mediated decay (NMD). This variant is also predicted to be deleterious by both CADD and FATHMM (score: 46.00 and 0.9922 with accuracy: 0.70 and 0.78, respectively). Additional analysis with the Alamut software predicted possible NMD of the mRNA.

Further Sanger sequencing of the c.5941C>T variant in the patient’s family confirmed the absence of mutation in both parents, indicating its *de novo* origin and thus supporting its pathogenicity (Fig. S1A in Supplementary Material).

## Discussion

MRFACD is a genetic disorder caused by molecular defect of the *MED13L* gene. To date, more than 70 patients with mutations in this gene have been identified, mostly in the past 2 years.^5^ It appears that the *MED13L* gene mutations are one of the most frequent causes of syndromic ID; however, in our in-house database consisting of over 700 patients with various neurodevelopmental disorders, ID and specific dysmorphic features, we found only one that caused mutation.^2^ In all the cases, the identified single point variants in the *MED13L* gene predominated the loss-of function mutations (76% of all cases).^5^

In this study, we describe a patient with severe ID, developmental delay, absence of speech, dysmorphic features and hypotonia. Due to the presence of the *LARGE1* gene deletion identified by aCGH, congenital muscular dystrophy-dystroglycanopathy with mental retardation (OMIM # 608840) was first suspected in the patient. However, NGS analysis did not confirm the presence of additional pathogenic variants in the preserved copy of the *LARGE1* gene. Further investigation revealed *de novo* deleterious c.5941C>T variant in the *MED13L* gene, which is predicted to cause NMD of the transcript as a result of premature termination codon activation p.(Gln1981*).

Recently, Torring et al. have summarized clinical presentation of the disease in 69 patients with the *MED13L* gene mutations. Most of the disease features were present in our patient (Table 1), including common symptoms, but also less frequent findings like completely absent speech, self-destructive behaviour and strabismus. To our knowledge, there was only one case described by Muncke et al. with nearly absent speech in a girl aged 7 years with serious cardiac defect and a *de novo* heterozygous balanced translocation that interrupted the *MED13L* gene on chromosome 12q24.^3^

**Table 1:** Comparison of major clinical features and their frequency in patients with likely pathogenic variants in the *MED13L* gene and this study patient.

	Clinical feature	Number of patients	This study patient
Clinical findings	ID or DD	69/69	+(severe ID and DD)
	Speech delay	68/69	+(no speech)
	Hypotonia	46/66	+(axial hypotonia)
	Coordination problems	20/60	+(stereotypic movements, wide-based gait)
	Congenital heart defects	12/64	–
	Autistic features	16/69	–
	Abnormal brain MRI	26/58	+(lateral ventricles asymmetry, wider posterior part of the left ventricle)
	Seizures	10/63	–
	Behavioural problems	16/51	+(self-destructive behaviour – biting hands)
	Strabismus	3/24	+(convergent strabismus – esotropia)
Dysmorphic features	Depressed/flat nasal bridge	19/33	+(flat nasal bridge)
	Broad/prominent forehead	16/21	+(prominent frontal eminence)
	Bulbous nasal tip	50/67	+
	Upslanting palpebral fissures	26/65	+(slight epicanthic folds)
	Low seat ears	17/33	–
	Bitemporal narrowing	8/33	–
	Horizontal eyebrows	7/33	+
	Macrosomia	14/33	+
	Macroglossia	9/33	–

Hypotonia, speech delay and brain abnormalities in MRI are quite common among these patients (70%, 99% and 45%, respectively). Also, dysmorphic features such as bulbous nasal tip, depressed/flat nasal bridge, upslanting palpebral fissures and abnormal chin have been previously reported in patients with the *MED13L* haploinsufficiency syndrome.

## Conclusion

Our findings support the conclusion that the phenotype of patients with loss-of-function variants in the *MED13L* gene could be a clinically recognizable MRFACD syndrome, especially when ID is accompanied by development and speech delay and specific dysmorphic features. We further define the phenotype caused by deleterious variants of the *MED13L* gene, broadening the phenotypic spectrum with uncommon findings of absent speech, strabismus, self-destructive behaviour, and constipations and confirm low penetration of cardiac abnormalities in patients with MRFACD syndrome. However, due to some similarity to other syndromes with ID such as FG syndrome caused by *MED12* mutation, 1p36 deletion syndrome or 22q11.2 deletion syndrome and proved variable clinical expression, the use of NGS is recommended for disease differential diagnosis.^6^, ^7^

## Key points

- Pathogenic variants in *MED13L* are common in the autosomal dominant form of syndromic intellectual disability.
- MRFACD is characterized by clinical heterogeneity – our patient, besides intellectual disability, developmental delay and hypotonia, presented self-destructive behaviour, strabismus and complete absence of speech, findings that are not common in patients with *MED13L* mutations.
- As the intellectual disability is not a pathognomonic feature and MRFACD shares clinical characteristics with other ID syndromes, it seems that NGS-based analysis should be applied to make a definite diagnosis.

## Supplementary materials and methods

### Whole exome sequencing (WES) and data analysis

DNA was extracted from the peripheral blood using standard extraction methods. Exome capture was performed using Agilent SureSelect Human All Exon V6 (60Mb, ~21,000 genes) (Agilent Technologies, Santa Clara, California, USA). Sequencing was carried out with 100 bp paired-end reads on an Illumina HiSeq 1500 system (Illumina, Inc., San Diego, USA). Reads alignment and variant calling was performed with a Burrows–Wheeler Alignment Tool and processed further by a Genome Analysis Toolkit (GATK, Broad Institute, Cambridge, MA, USA).^1^ The detected variants were annotated using ANNOVAR and converted to MS Access format for final manual analysis. First, variants in the *LARGE1* gene were analysed due to the result of the aCGH analysis. Next the analysis of variants in known genes involved in the pathogenesis of ID and novel genes expressed in skeletal muscle and/or CNS was performed. The analysis focused on missense, nonsense, non-frame small deletions/insertions and splicing variants absent in ExAC, 1000Genomes, Exome Variant Server databases and our own (Warsaw Medical University, Institute of Mother and Child) exome database of >1000 Polish individuals and was predicted to be pathogenic by *in silico* analysis (PolyPhen2, SIFT, Combined Annotation Dependent Depletion (CADD), Mutation Assessor, the Likelihood-ratio test (LRT), FATHMM, Mutation Taster).

Selected potentially pathogenic variants were analysed in a proband and its relatives with Sanger sequencing using BigDye Terminator v3.1 Cycle Sequencing Kit (Life Technologies) according to the manufacturer’s instructions on Applied Biosystems 3500xL Genetic Analyzer (Life Technologies, Carlsbad, CA, USA). Primers (forward: 5′-TGTGAACAACCAGAGGAGGA-3′, reverse: 5′-TGGATGGTTGATGATGTTGG-3′) used for amplification and sequencing were designed using the Primer3 online tool.

### Reference

1. Płoski R, Pollak A, Müller S, Franaszczyk M, Michalak E, Kosińska J, et al. Does p.Q247X in TRIM63 cause human hypertrophic cardiomyopathy? Circ Res. 2014;114(2):e2-5. doi: 10.1161/CIRCRESAHA.114.302662.
